# RETRACTION: Effect of Percutaneous Vertebroplasty Versus Percutaneous Kyphoplasty on Post‐Operative Wound Pain in Patients With Osteoporotic Vertebral Compression Fractures

**DOI:** 10.1111/iwj.70404

**Published:** 2025-03-21

## Abstract

**RETRACTION:** SiX. , ShanD. , Huol. , HuY. , ZouC. , WangB. , CaoJ. , and WuW. , “Effect of Percutaneous Vertebroplasty Versus Percutaneous Kyphoplasty on Post‐Operative Wound Pain in Patients With Osteoporotic Vertebral Compression Fractures,” International Wound Journal 21, no. 3 (2024): e14745, 10.1111/iwj.14745.38484743 PMC10940009

The above article, published online on 14 March 2024, in Wiley Online Library (http://onlinelibrary.wiley.com/), has been retracted by agreement between the journal Editor in Chief, Professor Keith Harding; and John Wiley & Sons Ltd. Following an investigation by the publisher, all parties have concluded that this article was accepted solely on the basis of a compromised peer review process. The editors have therefore decided to retract the article. The authors did not respond to our notice regarding the retraction.

**RETRACTION:** X. Si , D. Shan , l. Huo , Y. Hu , C. Zou , B. Wang , J. Cao , and W. Wu , “Effect of Percutaneous Vertebroplasty Versus Percutaneous Kyphoplasty on Post‐Operative Wound Pain in Patients With Osteoporotic Vertebral Compression Fractures,” International Wound Journal 21, no. 3 (2024): e14745, 10.1111/iwj.14745.38484743 PMC10940009

The above article, published online on 14 March 2024, in Wiley Online Library (http://onlinelibrary.wiley.com/), has been retracted by agreement between the journal Editor in Chief, Professor Keith Harding; and John Wiley & Sons Ltd. Following an investigation by the publisher, all parties have concluded that this article was accepted solely on the basis of a compromised peer review process. The editors have therefore decided to retract the article. The authors did not respond to our notice regarding the retraction.

